# *hecd-1* Modulates *Notch* Activity in *Caenorhabditis elegans*

**DOI:** 10.1534/g3.114.015321

**Published:** 2014-12-31

**Authors:** Yunting Chen, Iva Greenwald

**Affiliations:** * Department of Biochemistry and Molecular Biophysics, Columbia University, College of Physicians and Surgeons, New York, New York 10025; † Howard Hughes Medical Institute, Columbia University, College of Physicians and Surgeons, New York, New York 10025; ‡ Department of Genetics and Development, Columbia University, College of Physicians and Surgeons, New York, New York 10025

**Keywords:** *C. elegans*, *glp-1*, *lin-12*

## Abstract

Notch is a receptor that mediates cell–cell interactions that specify binary cell fate decisions in development and tissue homeostasis. Inappropriate Notch signaling is associated with cancer, and mutations in Notch pathway components have been associated with developmental diseases and syndromes. In *Caenorhabditis elegans*, suppressors of phenotypes associated with constitutively active LIN-12/Notch have identified many conserved core components and direct or indirect modulators. Here, we molecularly identify *sel(ar584)*, originally isolated as a suppressor of a constitutively active allele of *lin-12*. We show that *sel(ar584)* is an allele of *hecd-1*, the ortholog of human HECDT1, a ubiquitin ligase that has been implicated in several different mammalian developmental events. We studied interactions of *hecd-1* with *lin-12* in the somatic gonad and with the other *C. elegans Notch* gene, *glp-1*, in the germ line. We found that *hecd-1* acts as a positive modulator of *lin-12*/*Notch* activity in a somatic gonad context—the original basis for its isolation—but acts autonomously as a negative modulator of *glp-1/Notch* activity in the germ line. As the yeast ortholog of HECD-1, Ufd4p, has been shown to function in quality control, and *C. elegans* HECD-1 has been shown to affect mitochondrial maintenance, we propose that the different genetic interactions between *hecd-1* and *Notch* genes we observed in different cell contexts may reflect differences in quality control regulatory mechanisms or in cellular metabolism.

Notch is a receptor that mediates cell–cell interactions during animal development. Virtually all of the core components and many modulators of Notch signaling were first identified through genetic analysis in *Caenorhabditis elegans* and *Drosophila* (Greenwald 2012; Greenwald and Kovall 2013). Mutant forms of Notch, as well as of other core components and modulators of the signal transduction system, have been associated with congenital human disease syndromes, cancer, or Alzheimer’s disease.

Notch is a type 1 transmembrane receptor protein that is essentially a membrane-tethered transcription factor (Greenwald and Kovall 2013). Ligand-binding leads to proteolytic cleavages that release the intracellular domain, which translocates to the nucleus and promotes the transcription of target genes. Core components of the signaling system include the proteases that mediate the cleavage events and a sequence-specific DNA binding protein generically called “CSL” that is part of the nuclear transcription activation complex.

Missense mutations in the ectodomain can mimic ligand-binding, leading to ligand-independent cleavage and release of the intracellular domain and, therefore, to constitutive activity. In *C. elegans*, such mutant forms cause cell fate transformations (Greenwald and Seydoux 1990; Greenwald *et al.* 1983). Similar missense forms of *NOTCH1* have been found in many patients with T-cell acute lymphoblastic leukemia (T-ALL), where they result in aberrant cell fate decisions and drive growth to contribute to oncogenesis (Weng *et al.* 2004; Tzoneva and Ferrando 2012). Reducing the activity of components involved in the cleavage events or in the nuclear complex is an effective way to reduce constitutive signaling and has been the basis for genetic screens in *C. elegans* (Dunn *et al.* 2010; Katic *et al.* 2005; Levitan and Greenwald 1995; Tax *et al.* 1997).

In *C. elegans*, genetic analysis of potential components and modulators of Notch involves the two *Notch* orthologs, *lin-12* and *glp-1* (Greenwald 2012; Greenwald and Kovall 2013). These genes have unique roles in some cell fate decisions and are functionally redundant for others (Lambie and Kimble 1991). In this study, we include genetic analysis of a cell fate decision in early gonadogenesis uniquely mediated by *lin-12*, and a role in germ-line proliferation uniquely mediated by *glp-1*.

The *C. elegans* somatic gonad has a single anchor cell (AC), which induces and organizes the vulva. Two cells of the developing somatic gonad have the potential to be the AC; interactions between them, mediated by LIN-12, result in one becoming the AC and the other becoming a ventral uterine precursor cell (VU). The process by which the cells resolve their fates is called the “AC/VU decision” and involves differential transcriptional regulation of *lin-12* and other feedback elements (Seydoux and Greenwald 1989; Wilkinson *et al.* 1994). In a *lin-12* null mutant, both cells become ACs; in “*lin-12(d)*” constitutively active missense mutants, both cells become VUs (Greenwald *et al.* 1983). Because the AC is required for vulval induction, *lin-12(d)* mutants lack a vulva and are egg-laying-defective.

The germ line has a distal-to-proximal axis, with a mitotic zone in a distal region and a proximal zone in which the germline nuclei undergo meiosis and, further proximally, gametogenesis. Mitosis in the distal region is promoted by a ligand produced by the distal tip cell, which activates GLP-1 in the underlying germ line. Mutations that cause strong constitutive *glp-1* activity result in a “Tumorous” (Tum) phenotype, in which germ cells always remain in the mitotic cycle (Berry *et al.* 1997). In contrast, in the absence of *glp-1*, germline stem cells do not proliferate (Austin and Kimble 1987; Priess *et al.* 1987).

The allele *sel(ar584)* was originally identified as a suppressor of the Vulvaless phenotype of a *lin-12(d)* mutant (Katic *et al.* 2005). We molecularly identify *sel(ar584)* as an allele of *hecd-1*, the ortholog of the human HECDT1 ubiquitin ligase. We show that *hecd-1* behaves as a positive regulator of *lin-12*/Notch in the AC/VU decision and as a cell-autonomous negative regulator of *glp-1*/Notch in germline proliferation. We propose that the different genetic interactions reflect a difference in cell context between the somatic gonad and germ line.

## Materials and Methods

### Strains and genetic analysis

*Caenorhabditis elegans* var. Bristol strain N2 was the wild-type parent strain of all mutants and markers used. All strains were grown using standard procedures at 20°, except for strains containing *glp-1(ar202)* and *glp-1(bn18)* background, which were maintained at 15°. For strains that were scored at 23° or 15°, animals were maintained and handled at the temperature of interest prior to scoring. Key strains used are listed in Table 1.

**Table 1:** Strains analyzed in this study

**Strain Name**	**Genotype**
GS3347	*unc-36(e251) lin-12(n302)*; *hecd-1(ar584)*
GS5680	*glp-1(ar202)*; *him-5(e1490)*
GS3328	*sel-7(n1253) unc-3(e151)*; *arIs51*
GS6393	*hecd-1(ok1437)* (allele reisolated from RB1319)
GS6154	*lin- 12(n302)*; *hecd-1(ok1437)*
GS6551	*glp-1(bn18)*; *hecd-1(ok1437)*
GS6552	*glp-1(bn18)* (scoring control for GS6551)
GS6704	*unc-36(e251) lin-12(n302)*; *hecd-1(ok1437)*
GS6748	*rhIs4[glr-1p*::*GFP + dpy-20(+)]*; *hecd-1(ar584)*
GS6749	*rhIs4[glr-1p*::*GFP + dpy-20(+)]*; *hecd-1(ok1437)*
GS6750	*lin-12(n302)*; *hecd-1(ok1437)*; *him-5(e1467)*
GS6751	*unc-32(e189) lin-12(n676n930) arIs131[lag-2p*::*yfp]*; *hecd-1(ok1437)*
GS6752	*unc-32(e189) lin-12(n676n930) arIs131[lag-2p*::*yfp]* (scoring control of GS6751)
GS6761	*unc-32(e189) arIs131[lag-2p*::*yfp]*; *hecd-1(ok1437)* (scoring control of GS6751)
GS6759	*glp-1(ar202)*; *hecd-1(ok1437)*
GS6760	*glp-1(ar202)* (scoring control of GS6759)
GS6765	*glp-1(ar202)*; *hecd-1(ar584)*
GS6766	*glp-1(ar202)* (scoring control of GS6765)
GS6767	*glp-1(bn18)*; *hecd-1(ar584)*
GS6768	*glp-1(bn18)* (scoring control of GS6767)
GS6808	*rrf-1(pk1417)*; *glp-1(bn18)*
GS6809	*glp-1(bn18)* (scoring control of GS6808)
GS4537	*rrf-1(pk1417)*; *glp-1(ar202)*

“Scoring control” indicates that a *hecd-1(+)* strain was segregated from the same heterozygous genetic background used to generate the comparison with a *glp-1* mutant strain indicated.

### Whole genome sequencing and data analysis

The strain GS3347 containing *sel(ar584)* mutation was backcrossed four times with N2 before deep sequencing. Genomic DNA library of GS3347 was prepared following Illumina’s WGS sample preparation manual. Paired-end library preparation, sequencing, and base calling were performed according to the manufacturer’s recommendations through Illumina’s FastTrack Sequencing Services Laboratory. Initial sequence data were mapped to the sequence of wild-type N2 reference genomic sequence using Illumina Genome Analyzer. Further data analysis was performed with MAQGene using general parameters previously described (Bigelow *et al.* 2009).

The lesion associated with *hecd-1(ar584)* was verified by performing PCR, followed by Sanger sequencing. The sequence of *hecd-1(ok1437)* was determined by Sanger sequencing using primers flanking the predicted deletion region.

### Design of RNAi constructs for *C. elegans*

RNAi constructs targeting the HECT domain encoding region of *C34D4.14* was designed using a web tool E-RNAi version 3.0 (http://www.dkfz.de/signaling/e-rnai3//). Primer pairs for HECT domain were yc-334 (AAAAACCGGTAGTTCAAGAATTGGCCTGGA) and yc-335 (AAAAGGTACCTTCTTGGTTGCTTCACATTCC). Target regions were amplified and cloned into vector pL4440 (Addgene). Each construct was confirmed by Sanger sequencing and thereafter transformed into *Escherichia coli* strain HT115(DE3).

### RNAi experiments

Feeding RNAi experiments were performed at 20° as described (Timmons and Fire 1998). Briefly, gravid adults were bleached and the eggs were placed on plates seeded with HT115 cells expressing the dsRNA targeting the region of *hecd-1* encoding the HECT domain. A clone corresponding to the HECT domain, which should target all predicted isoforms, was used for the experiments shown in Figure 3 and Figure 4. T7 polymerase expression in the HT115 cells had been induced with 6 mM IPTG for at least 4 hr at room temperature before plating the eggs. To score the Pro or Tum phenotype at the adult stage, animals were DAPI-stained and scored 3 d after eggs were placed on plates.

### Imaging

All microscopy performed on live animals was performed on a Zeiss Axioplan2 microscope, with a consistent exposure time used for each marker assayed.

## Results

### *sel(ar584)* is an allele of *hecd-1*

**Figure 1:**
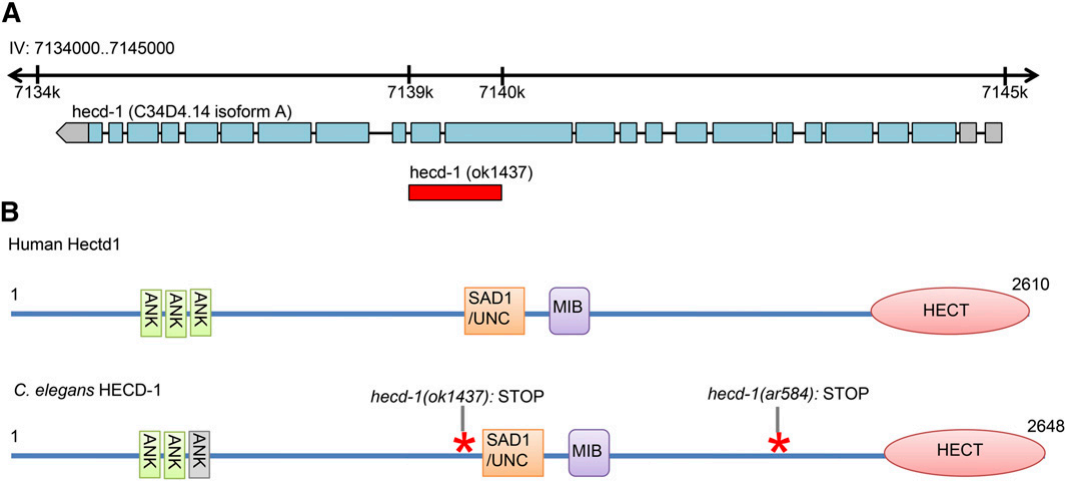
*C. elegans* HECD-1, its human ortholog, and mutations. **(**A) Genomic structure of *C. elegans hecd-1* (*C34D4.14*) isoform a. The deletion identified in *ok1437* causes a frameshift and results in a stop codon in all predicted isoforms. (B) Domain structure of *C. elegans* HECD-1, its human ortholog, and mutations. Using the sequence analysis protein SMART (Letunic *et al.* 2014), the human Hectd1 protein is predicted to contain three Ankyrin repeats, a SAD1/UNC domain, MIB domain, and HECT domain. The *C. elegans* HECD-1 isoform a is predicted by SMART to have similar domain architecture as human Hectd1, except the third Ankyrin repeat (gray) is slightly below threshold. The predicted stops associated with the *hecd-1(ar584)* point mutation and the *hecd-1(ok1437)* deletion mutation are indicated. Prior to the stop codon in *hecd-1(ok1437)* is a 78-amino-acid frame shift caused by the deletion mutation.

We performed whole genome sequencing of strain GS3347 to identify *sel(ar584)*. On LG IV, where *sel(ar584)* had been mapped (Katic *et al.* 2005), we identified a single predicted premature stop mutation in the *hecd-1* gene (Figure 1). *C. elegans* HECD-1 is the ortholog of yeast Ufd4p and of human HECTD1 (Shaye and Greenwald 2011). There are eight predicted isoforms, ranging in length from 2607 to 2650 amino acids (Harris *et al.* 2014). All isoforms contain a carboxy-terminal HECT ubiquitin ligase domain, and the stop codon associated with *sel(ar584)* is predicted to truncate all eight isoforms before the HECT domain (Figure 1). The domain structure of isoform a (2648 amino acids) is shown in Figure 1, as is the domain structure of a comparable isoform of its human ortholog.

**Figure 2:**
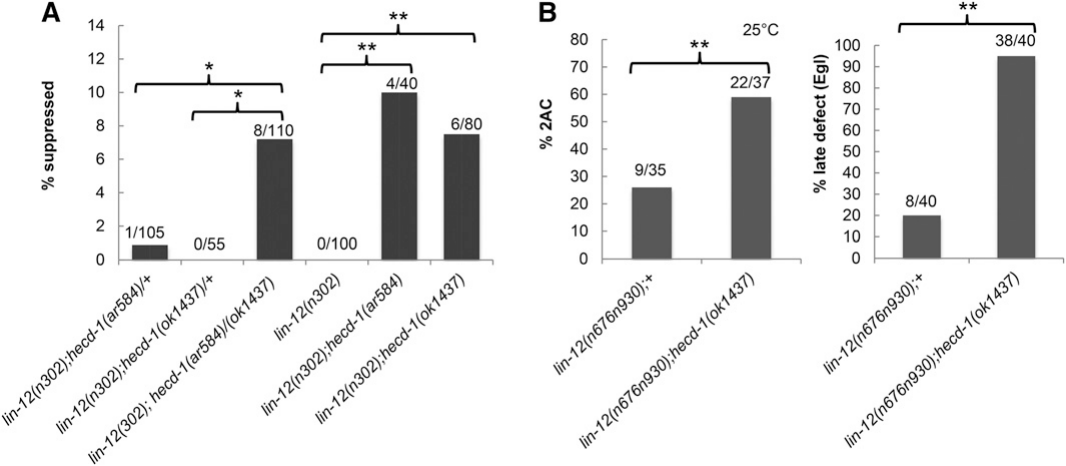
Genetic interactions between *hecd-1* and *lin-12*. Here and in all other figures: *significant P-value from Fisher’s test is <0.05 *vs.* control, and ** P<0.01 *vs.* control. (A) The egg-laying defect of *lin-12(n302)*, a constitutively active allele (Greenwald and Seydoux 1990; Greenwald *et al.* 1983), is mildly suppressed by *hecd-1(ar584)* and *hecd-1(ok1437)* at 20°. Trans-heterozygotes for the two alleles fail to complement: mild suppression of *lin-12(n302)* egg-laying defect by *hecd-1(ar584)/hecd-1(ok1437)* is observed. For the first set of three strains, the full genotype on chromosome III is *unc-36(e251) lin-12(n302)/ + lin-12(n302)*. (B) *lin-12(n676n930)* behaves like a partial loss-of-function allele at 25° and causes a “2 AC defect” (left) and a “late Egl defect” (right) (Sundaram and Greenwald 1993). *hecd-1(ok1437)* enhances both of these defects, indicating that loss of *hecd-1* further reduces *lin-12* activity. The full genotype on chromosome III of all the strains is *unc-32(e189) arIs131[lag-2p*::*2nls-yfp*::*unc-54 3′UTR] lin-12(n676n930)*. *arIs131* marks the anchor cell.

We performed further genetic analysis using an available deletion allele, *hecd-1(ok1437)*, which is predicted to cause a more severe truncation in all eight HECD-1 isoforms (see *Materials and Methods* and Figure 1). The results support the conclusion that *sel(ar584)* is an allele of *hecd-1*. First, *hecd-1(ok1437)*, like *sel(ar584)*, is a recessive suppressor of the 0 AC-Egl defect of *lin-12(n302)*, a mutation that results in elevated *lin-12* activity. Second, the two mutations fail to complement for suppression (Figure 2A). Third, as described below, *hecd-1(ok1437)*, *sel(ar584)*, and *hecd-1(RNAi)* behave similarly in several genetic assays in combination with alleles of *lin-12* and/or *glp-1*. We now call this allele *hecd-1(ar584)*.

### *hecd-1* is a positive modulator of *lin-12*/Notch activity in the somatic gonad

Both alleles of *hecd-1*, the original *hecd-1(ar584)* suppressor mutation and the independent deletion allele *hecd-1(ok1437)*, are likely to be strong loss-of-function or null alleles. Genetic analysis using *hecd-1(ok1437)* indicates that *hecd-1* is a positive modulator of *lin-12* activity in the somatic gonad. In the AC/VU decision, *hecd-1(ok1437)* suppresses the 0 AC-Egl defect of *lin-12(n302)* (Figure 2A) and also enhances the 2 AC defect of a hypomorphic allele, *lin-12(n676n930ts)* (Figure 2B). *hecd-1(ok1437)* also enhances a different egg-laying problem associated with reduced *lin-12* activity, the “late defect” (Sundaram and Greenwald 1993). The late defect has a complex basis including aberrations in uterine and sex muscle development (Eimer *et al.* 2002; Newman *et al.* 1995) (Figure 2B); we did not characterize the cellular basis of this enhancement further.

### *hecd-1* is a negative modulator of *glp-1*/Notch activity in the germ line

To examine the effect of *hecd-1* on *Notch* activity in the germ line, we used *glp-1* alleles that increase or decrease activity. Mitotic proliferation in the distal region of the germ line is driven by *glp-1* activity, and strong constitutive *glp-1* activity results in a “Tumorous” (Tum) phenotype (Berry *et al.* 1997). The *glp-1(ar202ts)* allele is a milder variant constitutive allele, which at 25° causes a “Pro” phenotype: a zone of ectopic proximal proliferation due to elevated *glp-1* activity in cells that would otherwise be meiotic (Pepper *et al.* 2003).

**Figure 3:**
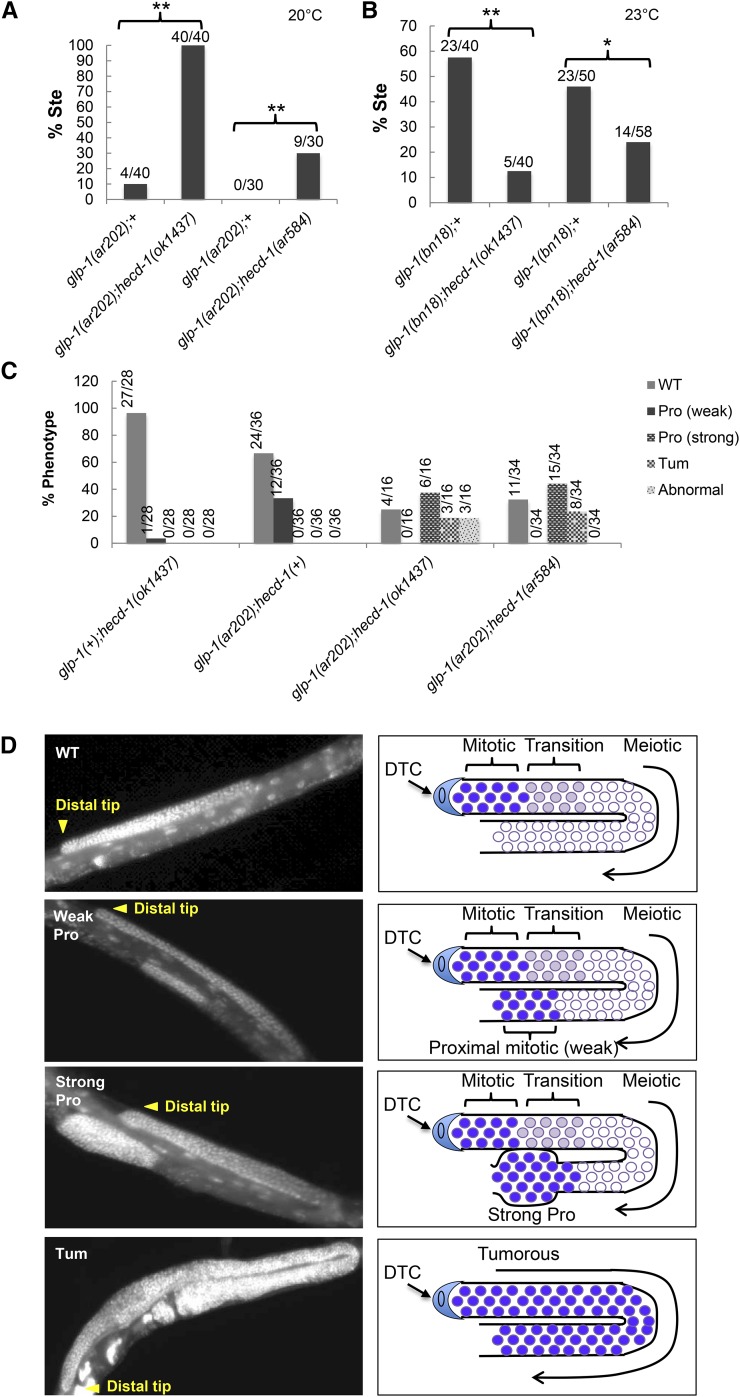
*hecd-1(ar584)* and *hecd-1(ok1437)* increase *glp-1* activity in the germ line. All experiments were performed at 20°. (A) *glp-1(ar202ts)* has elevated activity in the germ line, increasing mitotic proliferation and causing incompletely-penetrant sterility (Ste) at 20° (Pepper *et al.* 2003). Both *hecd-1(ar584)* and *hecd-1(ok1437)* enhance the penetrance of this defect, indicating that *glp-1* activity is increased. (B) *glp-1(bn18ts)* is a partial loss of function allele of *glp-1* (Kodoyianni *et al.* 1992). At 23°, *glp-1(bn18ts)* is ∼50% sterile, and its sterility is suppressed by *hecd-1(ar584)* or *hecd-1(ok1437)*. (C) DAPI staining reveals defects in germline anatomy. At 25°, *glp-1(ar202ts)* displays a “weak Pro” phenotype associated with mild elevation of *glp-1* activity (Pepper *et al.* 2003). Under our growth conditions at 23°, *glp-1(ar202ts)* display a mixture of weak Pro and wild-type. When *glp-1(ar202ts)* animals also carry *hecd-1(ok1437)* or *hecd-1(ar584)* and grown at 23°, a “strong Pro” and tumorous (Tum) phenotype is also evident, further supporting the inference that loss of *hecd-1* increases *glp-1* activity. (D) Images of *glp-1(ar202)*; *hecd-1(ok1437)* animals scored in (C), with cartoons depicting the interpretation of the phenotype. DTC, distal tip cell.

Because *hecd-1* behaves as a positive regulator in the somatic gonad, we were surprised to see that loss of *hecd-1* enhances the sterility of *glp-1(ar202)* at 20° (Figure 3, A–C), consistent with increased activity of *glp-1(ar202)*. To corroborate this inference, we examined the cellular basis of this phenotype in greater detail. We observed a range of phenotypes when either *hecd-1(ok1437)* or *hecd-1(ar584)* is combined with *glp-1(ar202)* at 20°, including a strong Tumorous phenotype associated with strong elevation of *glp-1* activity (Berry *et al.* 1997) and enhancement of ectopic proximal proliferation, the “Pro” phenotype, associated with milder elevations of *glp-1* activity (Pepper *et al.* 2003) (Figure 3D).

To see if this unexpected interaction was a property of *glp-1(ar202)*, we also combined *hecd-1(ok1437)* with *glp-1(bn18)* at 23°, a condition in which *glp-1* activity is partially reduced (Figure 3B) (Kodoyianni *et al.* 1992). We observed that loss of *hecd-1* suppresses the sterility associated with loss of *glp-1*, indicating that the interaction is not allele-specific. Thus, the results with both gain- and loss-of-function alleles indicate that *hecd-1* acts as a negative regulator of *glp-1* activity in the germ line, in contrast to the positive role it plays for *lin-12* for somatic cell fate decisions.

Maternally provided *glp-1* mediates many different decisions in the early embryo (Priess 2005), and loss of maternal *glp-1* activity results in embryonic lethality (Austin and Kimble 1987; Priess *et al.* 1987). We allowed hermaphrodites to reach fertility at the permissive temperature and lay eggs at the restrictive temperature: a higher proportion of *glp-1(bn18)*; *hecd-1(ok1437)* eggs than *glp-1(bn18)* eggs hatched [40/151(26%) *vs.* 7/83 (8%); *P* < 0.01]. This observation is consistent with *hecd-1* acting as a negative regulator of maternal *glp-1* activity. However, the hatched eggs arrested as L1 larvae, so embryonic development is still abnormal; we do not know the cellular basis of the improved rate of hatching observed.

### *hecd-1* acts autonomously in the germ line to modulate *glp-1* activity

**Figure 4:**
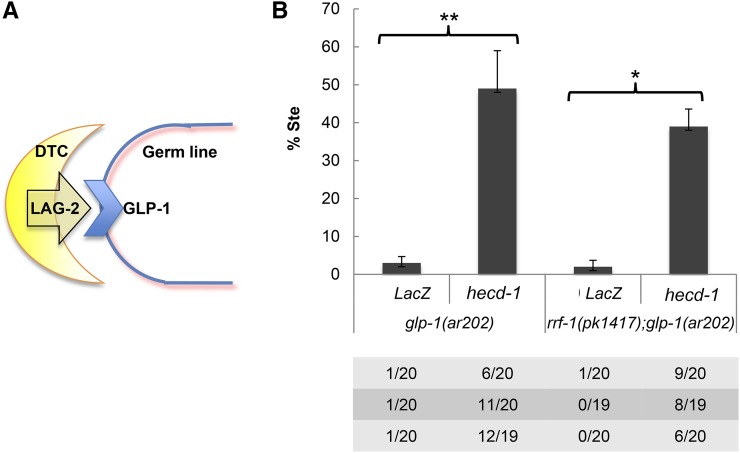
Evidence that loss of *hecd-1* acts autonomously in the germ line to increase *glp-1* activity. Bar graph shows the expression of mean±SE from three trials. (A) Cartoon depicting the cell–cell interactions: the somatic distal tip cell (DTC) is the origin of the LAG-2 signal that activates GLP-1/Notch in the germ line. (B) *hecd-1(RNAi)* enhances *glp-1(ar202)* sterility. Enhancement is still seen in the presence of *rrf-1(pk1417)*, a mutation that prevents RNAi in some somatic cells, including cells of the somatic gonad (Kumsta and Hansen 2012), indicating that *hecd-1* is likely to act to increase *glp-1(ar202)* in the germ line. There is no statistically significant difference between the values obtained in the *rrf-1(+)* and *rrf-1(pk1417)* background.

In the AC/VU decision, both interacting cells within the somatic gonad express ligand and receptor. However, for germline proliferation, the ligand-expressing distal tip cell of the somatic gonad and the receptor-expressing germline stem cells are distinct, making it more straightforward to determine the cellular focus of *hecd-1* activity for influencing *Notch* activity in this context. We asked whether *glp-1(ar202)* activity was increased by loss of *hecd-1* activity in the soma (signaling cell) or germ line (receiving cell) by comparing the effect of *hecd-1(RNAi)* in the background of *rrf-1(+)* or *rrf-1(pk1417)*, a mutation that preferentially eliminates RNAi in many somatic tissues, including the somatic gonad, without compromising RNAi in the germ line (Kumsta and Hansen 2012; Sijen *et al.* 2001). We found that *hecd-1(RNAi)* enhanced *glp-1(ar202)* regardless of the *rrf-1* genotype (Figure 4), suggesting that *hecd-1* acts autonomously in the germ line to modulate *glp-1* activity.

## Discussion

HECD-1 is the ortholog of human HECTD1 and yeast Ufd4p. We have identified *hecd-1* as a new modulator of Notch signaling in *C. elegans* with unusual genetic properties: loss of *hecd-1* leads to reduced *lin-12*/Notch activity in the AC/VU decision but increased *glp-1*/Notch activity in the germ line. We are unaware of any other modulator that has this distinctive genetic behavior. It is possible that the different genetic interactions reflect an intrinsic difference between LIN-12 and GLP-1. However the functional redundancy of LIN-12 and GLP-1 in several somatic cell fate decisions (Lambie and Kimble 1991) and the ability of GLP-1 to substitute for LIN-12 the AC/VU decision and other decisions uniquely mediated by *lin-12* (Fitzgerald *et al.* 1993) lead us to propose instead that the different genetic interactions we observed reflect differences in cell context that are not directly related to the Notch paralogs *per se*.

The yeast ortholog of HECD-1, Ufd4p, has been shown to be a quality control ubiquitin ligase (Hwang *et al.* 2010; Johnson *et al.* 1995; Ju *et al.* 2007; Ju and Xie 2006), and there appears to be substantial feedback regulation in the clearance of misfolded, aggregated proteins by quality control ubiquitin ligases including Ufd4p (Theodoraki *et al.* 2012). Because quality control is fundamental to eukaryotic cells, the conservation between HECD-1 and Ufd4p may reflect a conserved function in quality control for HECD-1 and at least some of the mechanisms that regulate it. *C. elegans hecd-1* was also recently identified in a screen for mutations that result in reduced ubiquitin-proteasome activity, and was further implicated in mitochondrial maintenance (Segref *et al.* 2014). The role in mitochondrial maintenance suggests a possible effect of loss of *hecd-1* on energy production or metabolism.

Different cell contexts may affect the way proteins fold or aggregate when misfolded, the dynamics or regulation of quality control mechanisms, or energetics. Thus, *hecd-1* may influence Notch activity indirectly through regulating one or more of these cellular properties. However, it is possible that HECD-1 acts directly, although at this level of genetic analysis we cannot know the target. Notch signaling involves many components, both membrane-associated and cytosolic, and many modulators, some of which are cell type-specific (Greenwald and Kovall 2013).

Many genes identified through genetic analysis in *C. elegans* have been proven to play similar roles in mammals. In mice, the ortholog HECTD1 has been shown to be a functional ubiquitin ligase required for normal craniofacial development (Sarkar and Zohn 2012; Zohn *et al.* 2007). Because aberrations in Notch signaling can also cause craniofacial abnormalities, we speculate that craniofacial abnormalities resulting from loss of HECTD1 may, at least in part, reflect effects on Notch signaling.
